# Effectiveness of Iodine-123 β-methyl-P-iodophenyl-pentadecanoic acid (BMIPP) Myocardial Scintigraphy for Cancer Therapeutics-Related Cardiac Dysfunction (CTRCD) in Breast Cancer Patients

**DOI:** 10.7759/cureus.25524

**Published:** 2022-05-31

**Authors:** Yuko Harada, Kyosuke Shimada, Yukino Kubota, Miyoko Yamashita

**Affiliations:** 1 Internal Medicine, Harada Naika Clinic, Kawasaki, JPN; 2 Cardiology, Kawasaki Municipal Ida Hospital, Kawasaki, JPN; 3 Breast Surgery, Kawasaki Municipal Ida Hospital, Kawasaki, JPN; 4 Palliative Medicine, Kawasaki Municipal Ida Hospital, Kawasaki, JPN; 5 Radiology, Kawasaki Municipal Ida Hospital, Kawasaki, JPN

**Keywords:** echocardiography, breast cancer, 123i-bmipp, myocardial scintigraphy, chemotherapy-related cardiac dysfunction (ctrcd)

## Abstract

Purpose: The optimal imaging modality for evaluating Cancer Therapeutics-Related Cardiac Dysfunction (CTRCD) other than echocardiography is currently not known. We conducted a retrospective study utilizing myocardial scintigraphy to detect early-stage CTRCD in asymptomatic breast cancer patients.

Patients and Methods: Fifty-five asymptomatic breast cancer patients who had received chemotherapy within three years were involved in this study. Echocardiography was performed for all patients before and during chemotherapy. Thallium (^201^Tl) and ^123^I-β-methyl-P-iodophenyl-pentadecanoic acid (^123^I-BMIPP) myocardial perfusion and metabolism scintigraphy were performed for all patients. Scintigraphy images were reviewed by several doctors including cardiologists, radiologists, palliative care physicians, and breast surgeons. The visual image assessment was then compared with the automated analysis utilizing Heart Risk View-S software (Nihon Medi-Physics Co Ltd, Tokyo, Japan). The results of scintigraphy were then compared with previous echocardiography data.

Results: Measuring global longitudinal strain (GLS) was impossible in 51% of patients. Measuring left ventricular ejection fraction (LVEF) was impossible in 15% of patients. A significant reduction of ^123^I-BMIPP uptake was observed in 15 patients out of 55 patients (27.3%). Among the 51 patients who were not previously diagnosed with CTRCD, 11 patients (21.6%) showed a significant reduction of ^123^I-BMIPP uptake.

Conclusion: Myocardial scintigraphy with ^123^I-BMIPP detected myocardial damage in asymptomatic patients. If echocardiography is difficult to perform, myocardial scintigraphy could provide a second option for evaluating CTRCD.

## Introduction

As chemotherapy has advanced in recent years, 10-year survival rates of breast cancer patients have become as high as 79.3% [1]. The mortality rate in Japan is highest for cancer followed by heart disease. Therefore, it has become a serious problem that cancer survivors may die of heart disease caused by cancer treatment. Cardiovascular disease caused by cancer treatment is called cancer therapeutics-related cardiac dysfunction (CTRCD) which has become a focus of worldwide attention leading to the academic field of Onco-cardiology. Guidelines of CTRCD have yet to be established; thus, the European Society of Cardiology (ESC) Position Paper published in 2016 and the American Heart Association (AHA) statement published in 2019 are serving as guidelines [2-3].

Currently, the first choice of imaging tool for diagnosis and follow-up for CTRCD is echocardiography [2-3]. To date, the worldwide consensus is to define CTRCD as a decrease in the left ventricular ejection fraction (LVEF) of >10 percentage points, to a value of <53% (normal reference value for two-dimensional echocardiography) [4]. In this expert consensus reported in 2014, global longitudinal strain (GLS) was considered to be the optimal parameter for early detection of sub-clinical LV dysfunction, and a relative percentage reduction of GLS of >15% from baseline is regarded as abnormal [4]. However, echocardiography may become a mental burden to patients after mastectomy. Echocardiography after mammaplasty is sometimes difficult or impossible because ultrasound is interrupted by implants. Echocardiography is also difficult in patients with chest deformity, obesity, emphysema, tight intercostal spaces, and heavily calcified ribs [5]. Shortfalls in echocardiography are caused by constraints due to dependency on acoustic windows and variable operator skills [5]. Sometimes echocardiography is suspended due to technical reasons and/or psychological reasons.

Thallium (^201^Tl) and ^123^I-β-methyl-P-iodophenyl-pentadecanoic acid (^123^I-BMIPP or iodine-123 BMIPP) myocardial perfusion and metabolism scintigraphy (a.k.a.., Tl/BMIPP dual-isotope myocardial scintigraphy) is the simultaneous scintigraphy of myocardial perfusion using ^201^Tl and myocardial metabolism using ^123^I-BMIPP. Both isotopes are administered by consecutive IV injections. The procedure is typically completed in less than one hour. There are no other side effects except for radiation exposure. The patient’s physical or psychological burden is, therefore, minimal. Myocardial scintigraphy offers better reproducibility and less interobserver- and intraindividual variability than echocardiography [5-7]. Thus, myocardial scintigraphy is more reliable than echocardiography for objective evaluation without human errors among operators. Issues with scintigraphy include radiation exposure and cost as compared to echocardiography. Previous studies in Japan reported that ^123^I-BMIPP myocardial dynamic single-photon emission computed tomography (SPECT) was useful in the early detection of doxorubicin- and taxan-induced cardiomyopathy, although the sample sizes were rather small [8-9].

In this study, Tl/BMIPP dual-isotope myocardial scintigraphy was performed in non-symptomatic breast cancer patients who received chemotherapy within three years. The aim of this study was to investigate whether myocardial scintigraphy could detect early-stage CTRCD, especially for patients who were not able to or wished to undergo echocardiography. To our knowledge, this is the largest single-center research on CTRCD using Tl/BMIPP dual-isotope myocardial scintigraphy.

## Materials and methods

Eligible patients with histologically confirmed breast cancer who received chemotherapy from November 2018 to November 2021 were approached for study participation by their treating oncologist and cardiologist at Kawasaki Municipal Ida Hospital in Kawasaki, Japan. All the patients provided written consent to be enrolled in this study. Regulatory approval for this study was granted by the research ethics board at Kawasaki Municipal Ida Hospital.

**Figure 1:**
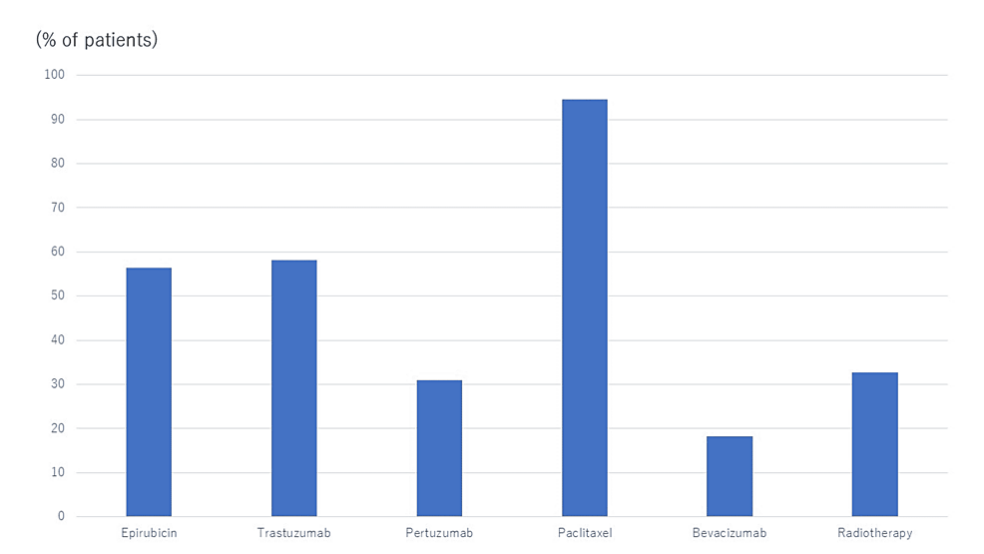
Therapies for breast cancer patients. Percentage of patients who received each therapy/therapies.

Medicine for chemotherapy was given in accordance with the guidelines of the Japanese Breast Cancer Society [10]. Asymptomatic patients who underwent multidrug therapy within three years were involved in this study. The commonly used anti-cancer drugs were Epirubicin, Trastuzumab, Pertuzumab, Bevacizumab, and Paclitaxel (Figure 1). All patients underwent echocardiography prior to chemotherapy and every six months during chemotherapy. Patients with known CTRCD diagnosed by the criteria of LVEF <53% and a decrease of LVEF by 10% were separated and compared with the other patients.

Patients fasted for over six hours prior to the study. They were injected with 111MBq of ^201^Tl, then immediately injected with 111MBq of ^123^I-BMIPP (Nihon Medi-Physics Co. Ltd, Tokyo, Japan) intravenously. SPECT images were acquired starting from 15 min after tracer injection using a digital gamma camera Symbia E (Canon Medical Systems Corporation, Tochigi, Japan).

Myocardial SPECT images were analyzed by an automated program called Heart Risk View-S software (Nihon Medi-Physics Co. Ltd., Tokyo, Japan). The software generated polar maps from myocardial SPECT data that are divided into 17 segments based on the guideline of the American Heart Association to calculate the mean count in each segment [11]. Following its former generation Heart Score View, these mean counts were compared with the normal ^201^Tl and ^123^I-BMIPP database developed for Japanese patients by the Japanese Society of Nuclear Medicine working group [12-14]. The mean percentage uptake in each segment was thereby calculated accordingly, converted to scores using a five-point scale ranging from normal to absent (0, normal; 4, absent), and then described on polar maps. Segments with mild, moderate, or severe reductions were defined as 60%-70%, 50%-60%, and 40%-50% uptake, and scored as 1, 2, and 3, respectively. Segments without uptake were defined as less than 40% uptake and were scored as 4. Normal segments were scored as 0 [14].

Charts of all patients were reviewed and echocardiography data (LVEF and GLS) were collected. The relative change of GLS (%∆GLS) was calculated as follows:

%∆GLS = (GLS after chemotherapy - GLS before chemotherapy)/GLS before chemotherapy

## Results

**Figure 2:**
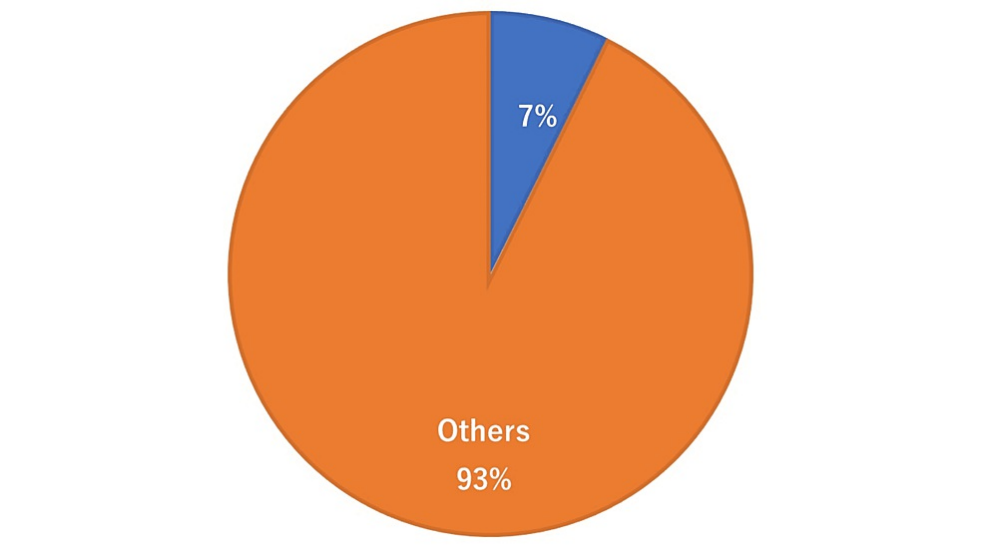
Patients with known CTRCD. CTRCD, cancer therapeutics-related cardiac dysfunction

Fifty-five patients were enrolled and their mean age was 62. Four out of 55 (7%) patients were previously diagnosed with CTRCD as shown in Figure 2.

**Figure 3:**
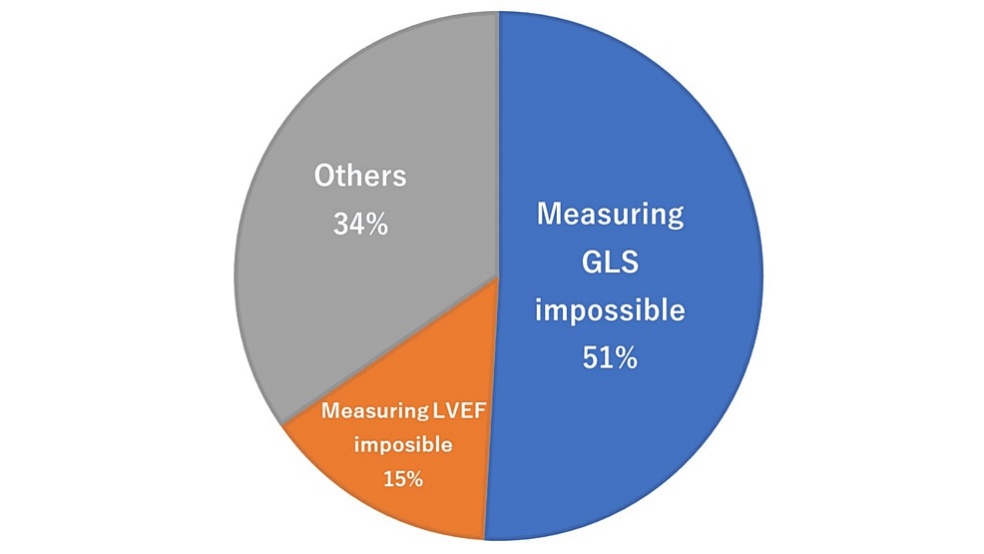
Patients with difficulty in measuring GLS and/or LVEF. GLS, global longitudinal strain; LVEF, left ventricular ejection fraction

Echocardiography, especially measuring GLS, proved difficult in 28 patients out of 55 (51%) as shown in Figure 3. Echocardiography was impossible to perform in eight patients out of 55 (15%) due to surgical wounds, scars, and other physical deformations.

**Figure 4:**
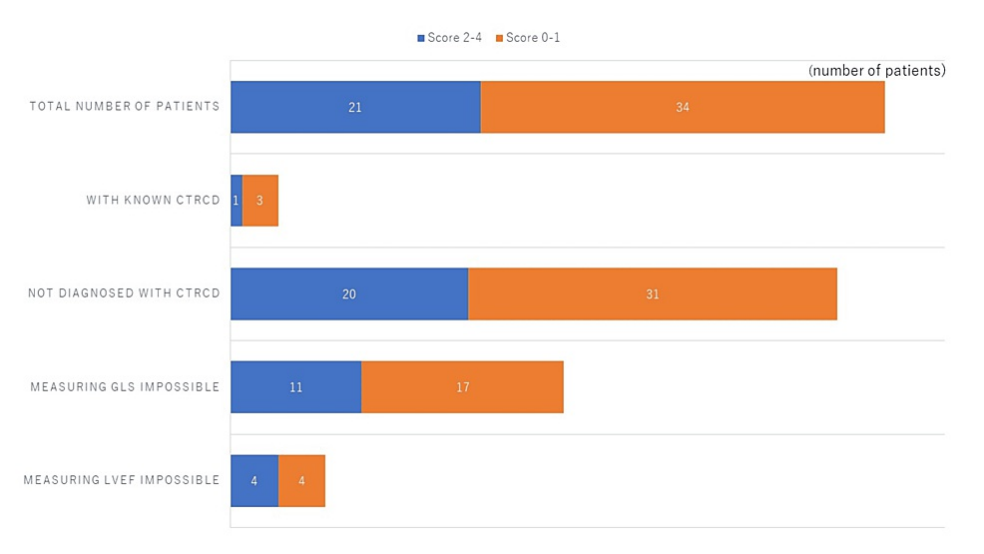
Reduction of thallium-201 uptake. The numbers of patients who showed significant reduction of ^201^Tl uptake are shown in blue bars. The rest of the patients (orange bars) in each category showed mild reduction or no reduction of ^201^Tl uptake.

**Figure 5:**
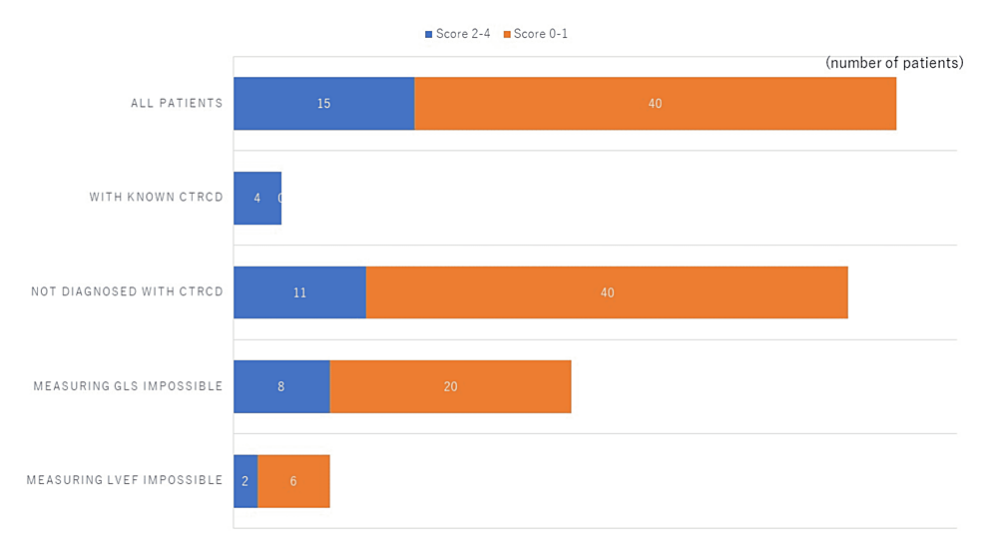
Reduction of iodine-123 BMIPP uptake. The numbers of patients with a significant reduction of ^123^I-BMIPP uptake are shown in blue bars. The rest of the patients (orange bars) showed mild or no reduction of ^123^I-BMIPP uptake. ^123^I-BMIPP, Iodine-123 β-methyl-P-iodophenyl-pentadecanoic acid

Heart Risk View-S scores of 2 and higher indicate significant reductions of ^201^Tl or ^123^I-BMIPP uptake. The numbers of patients with significant isotope uptake are listed in Figures 4-5.

A significant reduction of ^123^I-BMIPP uptake was observed in 15 patients out of 55 patients (27.3%) as shown in Figure 5. All patients with known CTRCD showed a significant reduction of 123I-BMIPP uptake. Among 51 patients who were not previously diagnosed with CTRCD, 11 patients (21.6%) showed a significant reduction of 123I-BMIPP uptake.

Reduction of ^201^Tl uptake was observed in 21 patients out of 55 patients (38.2%) as shown in Figure 5. However, only one patient with known CTRCD showed a significant reduction of ^201^Tl uptake. Among 51 patients who were not previously diagnosed with CTRCD, 20 patients (39.2%) showed a significant reduction of ^201^Tl uptake. Myocardial SPECT segments with reduced ^201^Tl uptake did not match with segments with reduced ^123^I-BMIPP uptake.

**Figure 6:**
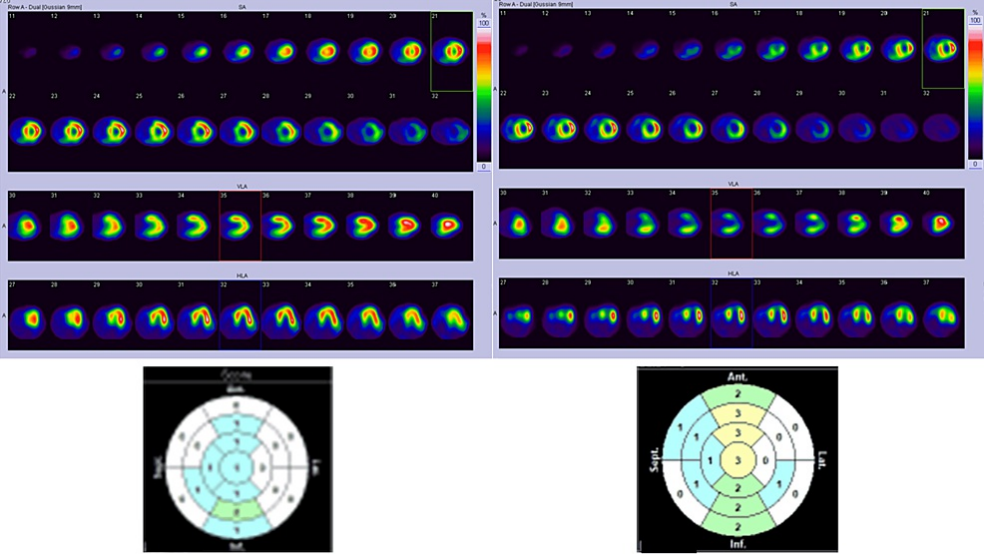
Tl/BMIPP dual-isotope myocardial scintigraphy of Case A. Top left: ^201^Tl myocardial SPECT. Top right: ^123^I-BMIPP myocardial SPECT. Bottom left: Heart Risk View-S scores for ^201^Tl uptake reduction displayed in 17-segments map. Bottom right: Heart Risk View-S scores for ^123^I-BMIPP uptake reduction displayed in 17-segments map. SPECT, single photon emission computed tomography; ^123^I-BMIPP, ^123^I-β-methyl-P-iodophenyl-pentadecanoic acid

One patient (Case A) was asymptomatic at enrolment but developed Levine III heart failure three months after scintigraphy (Figure 6). The other patients were non-symptomatic during the study.

**Figure 7:**
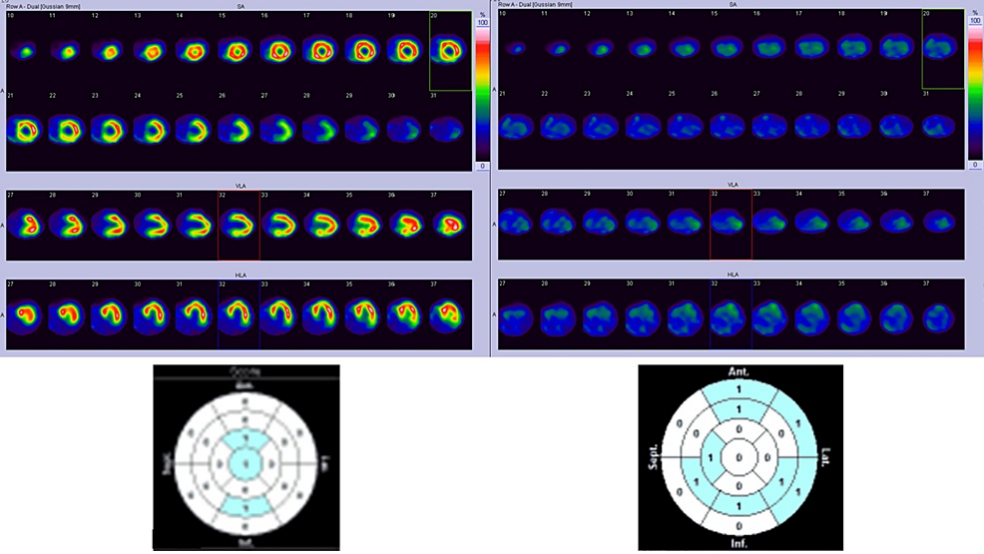
Tl/BMIPP dual-isotope myocardial scintigraphy of Case B. Top left: ^201^Tl myocardial SPECT. Top right: ^123^I-BMIPP myocardial SPECT. Bottom left: Heart Risk View-S scores for ^201^Tl uptake reduction displayed in 17-segments map. Bottom right: Heart Risk View-S scores for ^123^I-BMIPP uptake reduction displayed in 17-segments map. SPECT, single photon emission computed tomography; ^123^I-BMIPP, ^123^I-β-methyl-P-iodophenyl-pentadecanoic acid

Another patient (Case B) was diagnosed with CTRCD five months prior to the study. Case B revealed reduced LVEF from 71% to 48% during chemotherapy. Her dyspnea was resolved with discontinuation of chemotherapy and treatment including beta-blockers, but her ^123^I-BMIPP uptake was remarkably reduced (Figure 7). Heart Risk View-S software did not recognize the entire defect of ^123^I-BMIPP uptake, therefore, the scores were minimal as shown below.

**Figure 8:**
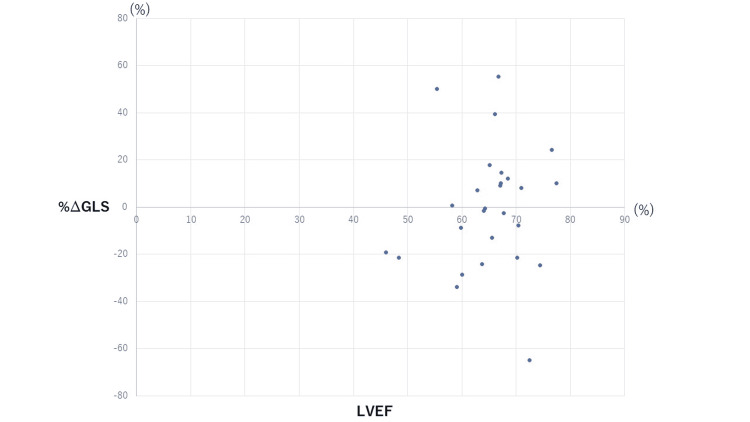
GLS and LVEF of the patients. The decrease ratio of GLS was calculated and compared with LVEF in a scatter plot. The decrease ratio of GLS was scattered over a wide range. GLS, global longitudinal strain; LVEF, left ventricular ejection fraction

The %∆GLS and LVEF did not reveal any relationship as exhibited in the scatter plot (Figure 8). In this study, LVEF ranged from 50% to 80% which is considered normal. However, %∆GLS ranged from -60 to +60. Pearson product-moment correlation coefficient was 0.09325 which is an indicator of non-correlation.

## Discussion

The Tl/BMIPP dual-isotope myocardial scintigraphy was successful in detecting abnormalities in asymptomatic breast cancer patients. A significant reduction of ^123^I-BMIPP is considered an early indicator of CTRCD. Diagnosis of CTRCD utilizing echocardiography, especially GLS, proved difficult in many patients in this study due to technical/physical issues. Myocardial scintigraphy, however, was welcomed by patients who experienced discomfort with echocardiography.

Thirty-eight percent of the patients exhibited a significant reduction of ^201^Tl uptake indicating reduced perfusion in the myocardium. However, none of the patients’ scores exceeded 3 or exhibited ischemic changes in the electrocardiogram. These patients may have experienced myocardial infarction with non-obstructive coronary arteries (MINOCA) or simply displayed artifacts that are often observed in anterior and inferior walls.

Many anti-cancer drugs are known to cause cardiotoxicity which includes direct effects not only on LVEF but also cardiac structure, diastolic function, arrhythmias, hypertension, systemic and pulmonary vascular function, thrombosis, and myocardial ischemia [15-16]. It has been well known that anthracyclines and Her2-targeted therapies cause CTRCD, but other anti-cancer drugs and radiation therapy may also cause CTRCD. In the present study, Epirubicin, Trastuzumab, Pertuzumab, Bevacizumab, and Paclitaxel were typically used. Epirubicin belongs to Anthracycline, while both Trastuzumab and Pertuzumab belong to Her2-targeted therapies, all of which are known to damage cardiomyocyte leading to heart failure [15-16]. Bevacizumab is a vascular endothelial growth factor inhibitor that may cause cardiomyocyte damage, hypertension, or coronary artery disease [15]. Paclitaxel belongs to taxanes of which cardiotoxicity was unexpected, however, arrhythmias are commonly observed and taxanes may contribute to cardiac dysfunction when used in conjunction with known cardiotoxic agents [15]. In the present study, all patients used multiple anti-cancer drugs in different resumes, which precluded a correlation of which drug affected ^123^I-BMIPP uptake. This should be elucidated in future studies.

The advantages of functional and myocardial perfusion scintigraphy are low intra- and inter-observer variability and high reproducibility [16]. The most commonly used techniques are planar multigated radionuclide angiography (MUGA) and ^123^I-metaiodobenzylguanidine scintigraphy (MIBG) which are acknowledged in position paper [2, 16]. However, a disadvantage of MUGA is its limited structural and functional information beyond LVEF [17]. The results of the present study suggest that ^123^I-BMIPP is suitable for detecting myocardial damage before LVEF starts to decrease. Alternative research compared ^123^I-MIBG and ^123^I-BMIPP scintigraphy indicating that myocardial imaging with ^123^I-MIBG could detect myocardial damage in the decreased heart-to-mediastinum ratio (H/M ratio) [18]. However, the H/M ratio does not reveal minor damage of the myocardium before LVEF decreases, plus they compared only 10 patients with normal controls.

There are some limitations of the present study. One study reported that fatty acid translocase (FAT)/CD36 gene mutation causes a total defect in ^123^I-BMIPP uptake and its prevalence was 0.47% (33/6,970) [19]. Another study reported the prevalence of CD36 deficiency was 0.3%-0.5% [20]. In patients with FAT/CD36 gene mutation, CTRCD cannot be detected by ^123^I-BMIPP scintigraphy. When we encounter a patient with a total defect in ^123^I-BMIPP uptake with no symptoms, we should also perform other cardiac imaging. Another limitation is that ^201^Tl myocardial scintigraphy picked up artifacts, especially from the implants.

Scores determined by Heart Risk View have proven to be favorably linear with standard visual interpretation by the specialists [14]. In this study, all the SPECT images were reviewed by cardiologists, radiologists, palliative care physicians, breast surgeons, and nuclear medicine technologists and were compared with scores calculated by Heart Risk View-S software. The evaluations by all doctors were linear with these scores except for Case B (Figure 7). In this case, the scores are not high for each segment in spite of remarkably reduced ^123^I-BMIPP uptake in SPECT image. Case B may have FAT/CD36 gene mutation which cannot be evaluated in our facility. Such scoring error occurred because this software scores by comparing each segment with the highest uptake lesion. Thus, it cannot score correctly when the entire heart shows reduced ^123^I-BMIPP uptake.

Myocardial scintigraphy was successful in determining myocardial damage in asymptomatic breast cancer patients. This provided an earlier sign of CTRCD prior to detection by echocardiography. A larger-scale multi-center prospective study is thus the next step.

## Conclusions

Myocardial scintigraphy with ^123^I-BMIPP proved successful in the early detection of myocardial damage in breast cancer patients. This revealed myocardial damage even though LVEF in echocardiography displayed normal. Compared with myocardial scintigraphy and echocardiography, early detection of CTRCD by measuring GLS in echocardiography turned out to be difficult. Therefore, for patients who experience difficulty with echocardiography, myocardial scintigraphy is a good alternative for the evaluation of CTRCD.
